# Implementing clinical education of medical students in hospital communities: experiences of healthcare professionals

**DOI:** 10.5116/ijme.5c83.cb08

**Published:** 2019-03-27

**Authors:** Åsa Alsiö, Berith Wennström, Björn Landström, Charlotte Silén

**Affiliations:** 1 Department of Infectious Diseases, Institute of Biomedicine, University of Gothenburg, Sweden; 2 Department of Anaesthesia/Surgery, Research and Development Center Skaraborg Hospital, Skövde, Sweden; 3 Skaraborg Institute for Research and Development, Skövde, Sweden; 4 Department of Learning, Informatics, Management and Ethics (LIME), Karolinska Insititutet, Stockholm, Sweden

**Keywords:** Community of practice, clinical education, medical students, interprofessional learning, reflection

## Abstract

**Objectives:**

To explore healthcare professionals’ experiences of implementing clinical education of medical students in communities of practice that previously focused on the delivery of healthcare services.

**Methods:**

Seven focus group interviews involving assistant nurses, nurses, and physicians were conducted at a regional hospital in Sweden. A total of 35 respondents participated. Open-ended questions were used to explore respondents’ experiences of medical students in their community. Data were analysed using qualitative inductive content analysis.

**Results:**

Three main themes emerged: Staff members becoming learners, structural and sociocultural changes due to the implementation, and features designing the settings of the implementation. Reflection and interactive learning processes among staff, patients, and students were found to stimulate individual learning, to improve the learning climate in the organisation, and to enhance the structure of the clinical work. Attitudes to education among staff members as well awareness of how education is organised appeared to be vital for their experiences and approaches.

**Conclusions:**

Implementing clinical education of medical students at a hospital previously focused on delivery of care was acknowledged to not only stimulate learning among staff but also trigger structural and cultural development in communities of practice. Opportunities for interprofessional interaction and reflection are vital to successfully implement a new student group in communities of practice. Addressing conceptions about and attitudes toward the clinical education of students among healthcare professionals are essential to promote their engagement in education.

## Introduction

Clinical education of medical students is of vital importance for our future healthcare. To meet the future societal needs of healthcare services, undergraduate medical programs have expanded in volume and numbers. The need for clinical education opportunities for an increasing number of medical students has outgrown the capacity of university hospitals. A more substantial portion of clinical practice must, therefore, take place in general hospitals. Although clinical training of different healthcare professionals is conducted in many of these hospitals, their main focus has traditionally been the delivery of healthcare services. From the educational authority and students’ horizons, this change in the location of undergraduate medical education raises questions about how to ensure the quality of clinical education and how to create a meaningful learning environment.

We argue that the attitudes and approaches of the healthcare professionals, in this study also referred to as staff, facing the new challenge of implementing education of undergraduate medical students in their practice are important to accomplish a successful implementation.^1^ A group of people engaging mutually in an activity using a range of methods of practice to reach a common goal, such as the delivery of healthcare, are defined as a community of practice.^2^ The climate in this practice influences individual healthcare professionals, the development of the local communities and also affects newcomers, students, and their education.^3^ A hospital is a community of practice, but it is also built up of smaller communities of practice within different wards or departments. Medical students will enter established communities of practice, a hospital with wards characterised by habits and activities with the primary goal of delivering efficient healthcare and not education. These communities develop in a process where its members participate in different activities share experiences and agree on the meaning of their experiences.^2^ How healthcare professionals in the hospital community interpret their experiences of the implementation will thus affect their future willingness to participate in the education of this group of students and potentially also the learning climate in their practices. In the current study the focus is on healthcare professionals and to obtain knowledge of how they perceive this new responsibility in their daily work.

The experiences of the staff responsible for handling a new group of students in their practice have previously not been investigated in depth. Few studies have explored how introducing a pedagogical practice, such as medical clinical education, will influence a hospital community at different levels, professionals, wards, and the hospital overall. A study examining staff attitudes towards teaching in a general hospital in England, in the context of the increasing number of medical students in clinical practice,^4^ found that the majority of respondents showed enthusiasm for teaching and experienced improved patient care and stimulation for learning among staff. Further, experiences of pressure were reported due to the competing demands of teaching and providing healthcare services. These competing demands were also reported in a study with senior academics and managerial staff in England.^5^ Although the studies mentioned above indicated that introducing medical education in new clinical settings can stimulate learning among individual staff, information about how and what staff members learn and how education influences different levels of a hospital organisation are scarce. The transformation process from being a hospital focused on healthcare production to becoming a teaching-oriented hospital can be expected to lead to changes and to require adjustment of individual staff members as well as the hospital organisation. Supporting students to achieve a number of specific learning goals necessitates engaging clinical tutors from among the staff with the capacity and time to facilitate the learning of students. Clinical training for a considerable number of students further requires physical space in clinical facilities, access to technical support, and opportunities for interaction among students, patients, and staff. Medical students’ arrival in a ward can thus be assumed to involve interaction with different levels at a hospital.

A macro-meso-micro level model^6^^,^^7^ may be used to elucidate influence and adjustment at different levels of an organisation. Taking a macro perspective means including the whole system, whereas only a part of a system is considered from a meso perspective and individuals are considered from a micro perspective.^6^ In this case, the hospital community represents the macro level, whereas the wards represent the meso level, and individuals from different professional groups represent the micro level. Although each different level constitutes a community of practice on its own, the macro-meso-micro level model suggests that the levels are interrelated and that communication flows across these levels.  An enhanced understanding of how the implementation is experienced, what it may lead to, and how it can be supported can be used to promote a meaningful learning climate in clinical settings. Exploring how the implementation of clinical education of medical students is experienced from the perspective of different categories of healthcare professionals is essential to develop knowledge of significant aspects that influence staff engagement and their learning. This study aims to explore healthcare professionals’ experiences of implementing education of medical students in communities of practice that previously focused on delivery of healthcare services.

## Methods

### Setting

In 2013, a substantial part of the undergraduate medical program was introduced at Skaraborg Hospital, a 670-patient-bed regional hospital serving 260,000 inhabitants. This was done in collaboration with the Sahlgrenska Academy at the University of Gothenburg and resulted in 30–40 medical students in clinical rotation per week at the hospital. Clinical education of fledgling assistant nurses, nurses, physiotherapists, and occupational therapists have been conducted at the hospital for decades. As part of preparing for the implementation of clinical education of undergraduate medical students at the hospital an organisation was formed with the purpose of supporting not only the learning processes of medical students but also different professionals to engage in and benefit from participating in these processes. To facilitate learning from experiences across different levels and departments in the organisation, an overarching coordinating team was formed, consisting of a project leader, a coordinator, and a responsible teacher from each of the four involved departments. The departments involved were the internal medicine, cardiology, anaesthesiology, and surgery departments. Teams of supervisors led by a responsible teacher were appointed for the involved departments. These teams had to develop learning activities for the students based on their respective learning objectives in collaboration with the course leaders at the university. The clinical supervisors were offered a 1.5 credit pedagogical course at the university. All healthcare professionals in the involved departments were to be informed of students’ learning objectives and the planned learning activities by the responsible teacher with the purpose of enhancing their engagement and participation in the education.

### Research approach

Exploring the experiences of introducing a new and more comprehensive clinical education program into several units of a hospital is a complex task. The implementation is neither uniformly nor unanimously perceived. To be able to support the development of a meaningful learning environment for students as well as healthcare professionals necessitates an increased understanding of plural perspectives rather than knowledge about the view of the majority. Considering the complexity of the research issue and the lack of distinct correct or incorrect answers, an explorative qualitative approach was employed.

### Data collection

A study using focus group interviews was initiated in spring 2014. Focus group interviews were chosen to facilitate interaction and capture a broad variety of views and to enable elaboration of different perspectives and thus construct new knowledge in dialogue with the research participants.

Two of the researchers, one physician (ÅA), and one nurse (BW) trained in qualitative methods conducted the focus group interviews. One of the researchers moderated the focus group discussion and the other took notes. All interviews were tape-recorded and began with a reflection on the open-ended question: ‘How have I experienced the introduction of medical students at my workplace?’ Initial answers were followed up with probing questions about participants’ expectations, how they and the workplace had been affected, and how they had handled the new situation. The participants were further encouraged to give examples of their experiences. The interviews lasted between 32 and 80 minutes and were transcribed verbatim by the researchers within one week of the interview sessions.

**Table 1:** Description of the focus groups in the study

Department	Profession	Number of Participants
Surgery/Anaesthesiology	Assistant nurses	7
Surgery/Anaesthesiology	Nurses	4
Surgery/Anaesthesiology	Physicians	5
Surgery/Anaesthesiology	Inter-professional group	6
Medicine/Cardiology	Assistant nurses	5
Medicine/Cardiology	Nurses	4
Medicine/Cardiology	Physicians	4

### Participants

All professional groups involved in the education of medical students physicians, nurses, and nurse assistants were included in the study. Eight focus group interviews were planned with six professionally homogeneous groups consisting of assistant nurses, nurses, or physicians, and with two multi-professional groups, mixing representatives from all three professions. To obtain rich data, purposeful sampling was adopted. Both men and women with variations in age and working experience were recruited from departments involved in the implementation of clinical education for medical students. Participants were recruited to all planned groups except to one of the multi-professional groups. Thus, the study consisted of seven focus groups of 4–7 persons, totalling 35 participants (Table 1). The age of the participants ranged from 27 to 67 years, and their working experience ranged from 6 months to 38 years. All the assistant nurses and nurses were women, but the group of physicians consisted of six men and five women. Permission to recruit participants for this study was obtained from the hospital management. Participants were given oral information in accordance with the provisions of the Ethical Review Act about the procedure of data collection, confidentiality, and voluntary participation before informed verbal consent was obtained from them^8^ The authors of the study complied with the ethical guidelines mentioned above.

**Table 2:** Illustration of the analysis process according to Graneheim and Lundman9

Meaning bearing unit	Code	Category	Sub-theme	Main theme
“… I believe my knowledge is reinforced when I have students and if there is something I do not know, I study and prepare to know the next time the issue comes up… it is an advantage to guide students to confirm your knowledge...”	Stimulated by demands of adequate knowledge	Perceived outcomes of the implementation	Implementation as stimulating reflective learning	Staff members become learners

### Data analysis

Qualitative content analysis was used for data analysis.^9^ The content was analysed in both its manifest form, that is, participants’ descriptions of their experiences, and its latent form, that is, the underlying meanings of participants’ expressions. To grasp the significance of the interview contents, the transcripts were read several times. Next, meaning-bearing units or sequences of importance were identified and further condensed and abbreviated into codes. Based on their variations and similarities, the codes were analysed and sorted into categories. The categories were interpreted after considering their underlying meaning, resulting in three main themes and six subthemes. The steps described above are exemplified in Table 2. Two of the authors read the entire material and identified meaning-bearing units, first separately and then together. All the authors of the current study participated in the latter part of the analysis to identify themes. Charmaz pointed out that saturation means that categories are ‘saturated’ when new data no longer provide new theoretical insights or reveal new properties.^10^ In the current study, the analysis of data was a sequential and simultaneous process that continued until new data could no longer add information to the emerging categories, and so-called saturation was achieved.

**Table 3:** Results presented as main and sub-themes

Main themes	Subthemes
Staff members becoming learners	Implementation as promoting interactive learning
	Implementation as stimulating reflective learning
Structural and sociocultural change due to the implementation	Implementation´s influence on structure
	Implementation´s influence on culture
Features designing the settings of the implementation	Conceptions of clinical education shape experiences
	Awareness is vital for engagement

## Results

The experiences of healthcare professionals following the implementation of clinical education for medical students are presented in the form of themes that reflect the latent content of the data. Overall, the results show that students’ introduction into the community of practice led to learning experiences among the staff and changes in the community of practice. In addition, the analysis of data exposed factors that were found to influence staff’s approaches to and experiences of participating in the implementation. Each of the three themes that emerged in the analysis, staff members becoming learners, structural and sociocultural changes due to the implementation, and features designing the settings of the implementation, is further clarified in the following sections. An overview of the themes and subthemes is presented in Table 3.

### Staff members becoming learners

All categories of healthcare professionals reported that the implementation of undergraduate medical education in the clinic led to situations that stimulated their own personal learning. Descriptions of how these situations stimulated their own learning constitute the core of this theme. Two mechanisms of learning were found to be important during the implementation of the clinical rotations: learning by interaction and learning by reflection. The following two subthemes, implementation as promoting interactive learning and implementation as stimulating reflective learning, illustrate how interaction and reflection stimulated learning among staff in situations related to the clinical education of medical students.

### Implementation as promoting interactive learning

Questions from students about patients and their diseases were described to stimulate consideration of new perspectives. Interactivity between students and different categories of staff in clinical situations were further found to promote learning through deep discussions about patients and their conditions. In the following quotes, interactions between students and staff were found to contribute to learning in different ways. Interactions where students asked questions created learning situations both for the staff and the students.

“… I believe it has been stimulating to talk with the students. They ask questions and come up with new perspectives that you have not thought about previously. You can be a bit caught up in your own perspective, but they can provide new ideas … well, you learn from each other…” (Physician)

Patients learned about their own condition when they listened to explanations meant for the students:

“… since everything is explained carefully for the medical students when the patient is present … it’s no doubt that the patient will be better informed about his condition …” (Nurse)

Discussions about diseases with the students was found to contribute to staff learning.

“… I learnt a lot. We have a lot of discussions about patients and their diagnosis. It takes much longer than an ordinary round. But still … it is positive…” (Assistant nurse)

### Implementation as stimulating reflective learning

Situations related to supervision were found to promote learning. Different teaching situations related to the education of medical students where the respondents experienced reinforcement or awareness of their own knowledge through reflection were described. In the following quote, student’s questions were found to stimulate reflection and bring the respondent´s knowledge to the fore.

“… I enjoy when I am being asked questions … when I reflect and answer I become aware that I know a lot. That’s inspiring…” (Assistant nurse)

Supervising students calls for reflection on how to support student’s learning and promotes awareness of tacit knowledge.

“…As a preceptor you need to think about things in a pedagogical way … Why you do things in a specific way. You do not do that when you are on your own … Its valuable for your own understanding…” (Nurse)

 Contact with students stimulates reflection on the respondent´s own knowledge and identification of the respondent’s own learning needs.

“… I believe my knowledge is reinforced when I have students, and if there is something I do not know, I study and prepare to know the next time the issue comes up … it is an advantage to guide students to confirm your knowledge...” (Physician)

### Structural and sociocultural change due to the implementation

Culture and structure at both the meso and macro levels of the hospital were found to be affected by the implementation. Influence on structure was found in smaller ward communities, whereas influence on culture was found in the larger hospital community. These changes are further illuminated in the following subthemes.

### Implementation’s influence on structure

The participating healthcare professionals stated that the implementation had an influence on the structure of the clinical work. In local ward communities, implementation was described as an adaption of clinical situations to enable students to participate in them and learn. The adaptive process to facilitate learning of students was also found to affect patients. For example, central aspects of importance for the care, diagnosis, and treatment of patients were reorganised and clarified for all involved actors subsequent to the implementation. In the following quote, a physician describes his experience of how implementation influenced the structure of clinical work in his clinical setting:

“… the flow of information is elucidated; which issues are important, what the treatment is and how the patient should be managed … the communication becomes stringent, everything is clear and well planned …” (Physician)

### Implementation’s influence on culture

Among the respondents, it was found that the introduction of medical students in clinical settings contributed to cultural changes within the hospital community. Cultural change was described as a more open climate where both patients and staff were free to ask questions. The implementation was described to reduce hierarchies between different professionals by increasing their contact and interaction. Additionally, learning was found to be given more focus. In the following quote, a nurse observed a pedagogical shift at the hospital as a result of the implementation, causing attitudes to supervision and pedagogy to be changed.

“… Guiding medical students had a positive impact at the hospital, pedagogic ideas are spread … the attitude to supervision in general has changed …” (Nurse)

### Features designing the settings of the implementation

Features related to awareness, expectations, and attitudes toward implementation among staff were found to affect staff’s approaches and experiences. Staff interpretations of implementation designed the setting for learning of both students and staff in the clinical communities. These features and their impact on behaviour and experiences are further clarified in the following subthemes.

### Conceptions of clinical education shape experiences

This subtheme explains how sociocultural factors such as attitudes and perceptions influence approaches to and experiences of the implementation. Attitudes toward clinical education had an impact on how staff behaved during the implementation process. Participation and engagement among staff were found to be enhanced by the attitude that clinical education is beneficial for oneself, the local community, or the larger hospital community.

Perceptions of clinical education as being a condition for future delivery of healthcare were found to motivate engagement, whereas perceptions of education as taking place at the expense of healthcare were found to hinder engagement. The view of education as a means of recruitment that motivates investment of time is found in the following quote.

“…To complete tasks while supervising takes a longer time. That’s not bad, that’s the way it is and must be, time must be allowed for guidance. In addition, it is a way for the hospital to promote itself and attract all kind of students …” (Nurse)

The following quote by a nurse offers another perspective of education’s negative impact on the delivery of care.

“…It’s time-consuming to educate, my regular clinical work is suffering… other colleagues have to do my job instead …” (Nurse)

The attitude that the implementation was taking place at the expense of learning opportunities for other professionals or groups was expressed in the following quote by a resident.

“… the problem is that they (the medical students) are always present, always standing in the fore… they (the medical students) are always asked first and residents miss interesting learning opportunities in the operating theatre since they (the residents) are not as easy to get in touch with and thus are not asked to participate.” (Physician)

Further, the perception of supervision was considered important to the approach to implementation. Defining the role as inspiring and or important for promoting recruitment emerged as an attitude that enhanced engagement in practice among the involved professionals. In the following quote by a physician, allocating time for explanation made him feel joyful.

“… I enjoy explaining. I normally take the time to explain how I think and plan. Now I do it more thoroughly, in a more reflective way, it takes a little longer, but it works out fine…” (Physician)

### Awareness is vital for engagement

Awareness of the roles and responsibilities of students and healthcare professionals were found to be important to how individual staff members interpreted their experiences. Awareness was found to support engagement, whereas lack of awareness was found to counteract engagement. In the following quotes, a physician and a nurse assistant share their experience of implementation and lack of awareness of their own roles and responsibilities or those of students.

“… When I was in the emergency ward, I did not really know what my role was and if I was supposed to be the responsible clinical mentor … the clinical guidance that I provided, was it all that was done? … it does not seem to be completely safe for the patients …” (Physician)

“… It is difficult to know who is who … we have new staff all the time, so I think … who is who, what are they allowed to do, what are they not allowed to do, who should I contact, who should I not contact, it is not obvious to me at all times…” (Assistant nurse)

Engagement in supervising medical students in clinical practice was stimulated by knowledge of what students are expected to learn and by having the time needed to engage in their learning. Supervision of students in clinical situations with allocated time and focus on teaching were explained to promote learning also among healthcare professionals. In the following quote, a supervisor describes how she engaged in and enjoyed educating medical students at her clinic, particularly when she was aware of students’ learning objectives and had reserved time for their education.

“… I enjoy having students at the outpatient clinic, I take the time to explain things … We have reserved time for it. I know their learning objectives. It is stimulating…” (Physician)

## Discussion

This study was conducted to expand the understanding of healthcare professionals´ experiences when introducing medical students into their community of practice. Overall, the results indicate that students’ introduction promoted learning at the macro, meso, and micro levels of the community. Experiences of stimulation to learn among individual staff members after the introduction of education of medical students echoes previous findings.^4^ However, in this study, implementation was found not only to support the development of individual healthcare professionals but also to influence the culture and the organisation of care in communities of practice. In addition, aspects of importance for change and learning in a community including how the professionals was stimulated to learn by the implementation, what they learned, how the community developed and which factors that were found to influence the motivation to participate in the implementation process emerged from the data in the current study. Interesting views arose regarding how the implementation contributed to learning among staff. Reflection and interaction between staff and students were enhanced as a consequence of the implementation, and these processes promoted learning. The interaction often came about while taking care of – and communicating with – patients, but it also came about in other encounters in the clinical work. Notably, the reflection was found to support a deeper understanding of experiences, which is in line with the educational literature’s argument that reflection on experiences is important for meaningful learning.^11^^-^^13^ It is also consistent with Harden’s ideas of reflection in clinical education as a tool for moving from knowing what the right thing to do is, to becoming aware of why it is the right thing to do.^14^

Reflection in groups with different categories of staff and students has previously been argued to promote the development of teamwork capabilities, which are considered an important aspect of competence in healthcare professionals.^15^ Interaction in general and interprofessional interaction, in particular, was in the current study found to be important mechanisms for learning among individual staff members. Further, the interaction was found to lead to a change in the learning climate and the structure of the clinical work. This corresponds to previous studies indicating that collaboration and the learning climate may be enhanced by interprofessional learning, which is defined as learners from two or more professions learning with, from, and about each other to improve collaboration and the quality of care.^16^^,^^17^

According to a phenomenographic perspective, the approach to participation among staff will determine what they learn from their experiences.^18^ Meaningful outcomes for individual staff members and the hospital community will thus be dependent on the adoption of an engaged approach. Interestingly, both attitudes to, and awareness of, the implementation emerged as vital to how the staff approached the implementation. Awareness of the roles and responsibilities of different staff and students was described as an important condition for an engaged approach; further, recognising clinical education as being beneficial for oneself or the community was also found to stimulate engagement. The contrasting views of education taking place at the expense of healthcare or one’s own development were found to elicit frustration and stress. Considering that, in this study, the implementation emerged as a process of change and learning that may have a beneficial impact on the entire community, it is essential to support staff to engage in the implementation and experience meaning. This requires both knowledge of determinants of motivation and strategies to influence them.

One notable sub-theme unearthed in this study was that of awareness as being vital for engagement; importantly, this may include different aspects of motivation. In the pedagogical literature, an individual´s motivation to engage in activities is thought to depend on his belief in having sufficient capacity to succeed and also the extent to which the process and goal are perceived to be meaningful for him.^19^ Lack of awareness of the different roles and responsibilities of involved actors within the sphere of clinical education may influence both the perception of capacity and meaning of participation among the involved staff. Interestingly, the emergence of attitudes towards clinical education as a stimulator or a hindrance for engagement reflects how motivation to participate is dependent on perceived meaning. Therefore, key objectives when implementing a new student group in a community of practice would be to promote awareness and experiences of meaning of participation among staff. Given that mutual engagement for reaching a common goal are required for the development of a community of practice 2, negotiating the goals and meanings of educating a new group of students in the community amongst the members of the community becomes essential. This study provides support for creating opportunities for staff to elaborate upon both the purpose and organization when implementing clinical education for new groups of students within the hospital community. Detailing purpose and organization may both clarify organizational issues and also address views that counteract engagement.

### Limitations of the study

The focus group interviews generated rich data. We consider that a strength of this study. There was active participation in all of the seven group discussions, and in six of the groups, the atmosphere was perceived as open and permissive. In one group, the participants interrupted each other several times, and this may have hampered the group dynamic. However, similar data were obtained for all seven groups.

The authors’ experience of supervising students in clinical practice, that is, a professional pre-understanding, may have affected the interactive process of collecting, analysing, and interpreting the data. However, no analysis is neutral. Interpersonal interactions make the researcher a part of his/her observations,^20^ thus perhaps introducing the risk of weakness in scientific rigor. Improving rigor in regard to such issues demands awareness and reflexivity. All authors were engaged in reflection at different stages of the study, and their different experiences made it possible for them to challenge each other’s assumptions and to continuously return to data for confirmation of interpretations as well as to reflect on methodological procedures. This investigator triangulation can be seen as a strength in regard to ensuring trustworthiness.^21^ The current qualitative study was carried out in one healthcare context. In this context, participants were varied with respect to their healthcare professions; additionally, the participants spanned a broad range of ages and work experience durations. Although the majority of the participants were women, this mirrors the demographics in most health service institutions. To enhance the credibility of the analysis, the results were presented to and discussed with the staff in all the wards involved in the study. The context, assumptions, participants, and data analysis method are described as clearly as possible to enable an assessment of the potential transferability of the results to other healthcare contexts. The results were also discussed in relation to theories allowing transfer to other contexts in a broader sense.^21^

## Conclusions

Implementing clinical education of medical students at a hospital previously focused on delivery of care was found to stimulate both individual learning among staff and trigger organisational change. Interprofessional interaction and reflection on experiences were found to be means for cultural and structural development of the community of practice when introducing new students into the practice. Motivation to participate in the implementation among staff was influenced by the awareness of ones’ roles and responsibilities within and during the implementation and by conceptions of education within the hospital community. These aspects of motivation need to be addressed for a successful introduction of new students in a community of practice. Considering the increasing number of medical students in need of clinical education opportunities in many countries, future studies of how to best support staff engagement when implementing education of medical students in communities of practice are warranted.

### Acknowledgements

The authors would like to acknowledge all the staff who agreed to participate in and contribute to the focus group interviews. Further, we would like to acknowledge Matilda Liljedahl, MD, PhD, Sahlgrenska Academy, University of Gothenburg, Beth Maina Ahlberg, Professor, Skaraborg Institute, Skövde, Sweden, and Jon-Emile S. Kenny, MD, MSc, Chief Medical Officer FloSonics Medical, Toronto, Canada, for their valuable comments on the manuscript.

### Conflict of Interest

The authors declare that they have no conflict of interest.
